# Transfer RNA Methyltransferases from *Thermoplasma acidophilum*, a Thermoacidophilic Archaeon

**DOI:** 10.3390/ijms16010091

**Published:** 2014-12-23

**Authors:** Takuya Kawamura, Ryou Anraku, Takahiro Hasegawa, Chie Tomikawa, Hiroyuki Hori

**Affiliations:** Department of Materials Science and Biotechnology, Graduate School of Science and Engineering, Ehime Univsersity, Bunkyo 3, Matsuyama, Ehime 790-8577, Japan; E-Mails: t.kwmr.0115@gmail.com (T.K.); corydoras.symbion@gmail.com (R.A.); x534065z@mails.cc.ehime-u.ac.jp (T.H.); tomikawa.chie.mm@ehime-u.ac.jp (C.T.)

**Keywords:** RNA modification, tRNA methyltransferase, archaea

## Abstract

We investigated tRNA methyltransferase activities in crude cell extracts from the thermoacidophilic archaeon *Thermoplasma acidophilum*. We analyzed the modified nucleosides in native initiator and elongator tRNA^Met^, predicted the candidate genes for the tRNA methyltransferases on the basis of the tRNA^Met^ and tRNA^Leu^ sequences, and characterized Trm5, Trm1 and Trm56 by purifying recombinant proteins. We found that the *Ta0997*, *Ta0931*, and *Ta0836* genes of *T. acidophilum* encode Trm1, Trm56 and Trm5, respectively. Initiator tRNA^Met^ from *T. acidophilum* strain HO-62 contained G^+^, m^1^I, and m^2^_2_G, which were not reported previously in this tRNA, and the m^2^G26 and m^2^_2_G26 were formed by Trm1. In the case of elongator tRNA^Met^, our analysis showed that the previously unidentified G modification at position 26 was a mixture of m^2^G and m^2^_2_G, and that they were also generated by Trm1. Furthermore, purified Trm1 and Trm56 could methylate the precursor of elongator tRNA^Met^, which has an intron at the canonical position. However, the speed of methyl-transfer by Trm56 to the precursor RNA was considerably slower than that to the mature transcript, which suggests that Trm56 acts mainly on the transcript after the intron has been removed. Moreover, cellular arrangements of the tRNA methyltransferases in *T. acidophilum* are discussed.

## 1. Introduction

*Thermoplasma acidophilum* is a thermoacidophilic archaeon that grows optimally at 59 °C and pH 1.9 [1]. The characteristic property of this archaeon is that the cells are very irregular in shape due to the lack of a cell wall [2,3]. Despite this, the cytoplasmic membrane tolerates an acidic environment at high temperatures. Consequently, components of the membrane have been studied in detail [4]. Furthermore, lipoylation of proteins [5], biosynthesis of lipids [6], cell surface glycoproteins [7] and a channel protein [8] have been also studied in *T. acidophilum*. Given that a prokaryotic histone-like DNA binding protein was discovered first from *T. acidophilum* [9,10], the bacterium has been used as a model system to investigate DNA replication, DNA repair, and transcriptional initiation in archaea [11,12,13,14,15,16]. Furthermore, the energy metabolism of *T. acidophilum* has been studied in detail because it can grow under an extreme microaerophilic environment [17,18]. Genome sequencing elucidated that the *T. acidophilum* genome encodes only approximately 1500 open reading frames [19]. Consequently, large protein complexes such as the proteasome and chaperonin are composed of a relatively limited number of protein subunits, and thus they have been studied and compared with their more complicated counterparts from eukaryotes [20,21,22,23].

**Figure 1:**
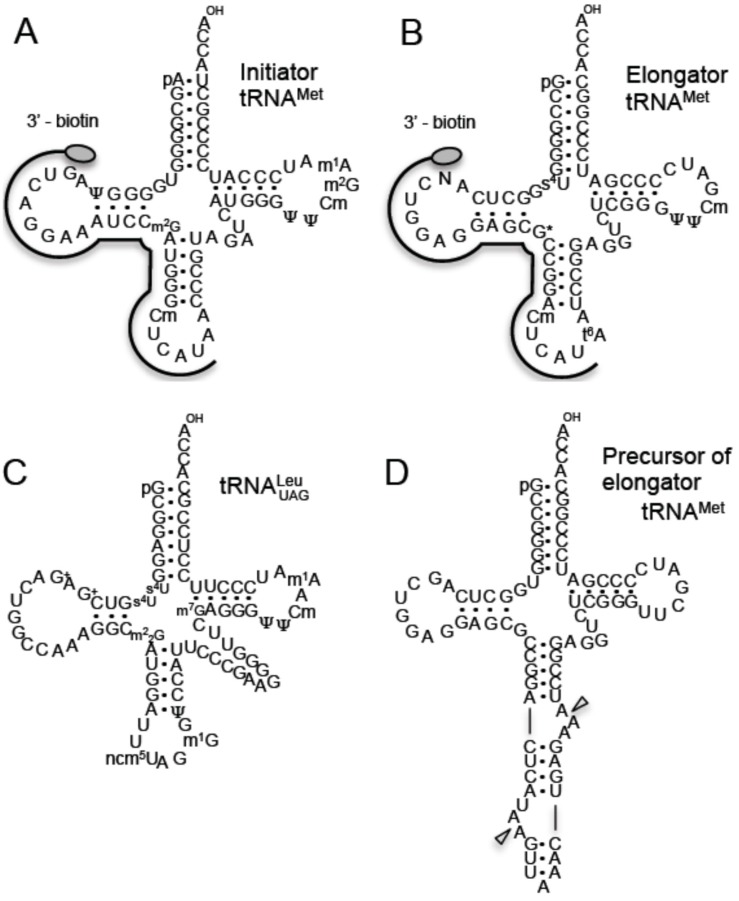
tRNAs and precursor tRNA from *T. acidophilum*. The sequences of initiator tRNA^Met^ (**A**), elongator tRNA^Met^ (**B**) and tRNA^Leu^_UAG_ (**C**) are depicted by cloverleaf structures. The regions to which the 3’-biotinylated DNA probes hybridize are illustrated. The abbreviations are as follows: pseudouridine, ψ; *N*^2^-methylguanosine, m^2^G; 2'-*O*-methylcytidine, Cm; 1-methyladenosine, m^1^A; 4-thiouridine, s^4^U; unknown modification, N; unidentified G modification, G*; *N*^6^-thereonylcarbamoyladenosine, t^6^A; archaeosine, G^+^; *N*^2^,*N*^2^-dimethylguanosine, m^2^_2_G; 5-carbamoylmethyluridine, ncm^5^U; 1-methylguanosine, m^1^G; 7-methylguanosine, m^7^G. The precursor of elongator tRNA^Met^ contains an intron at the canonical site (**D**). Triangles show the cleavage sites of tRNA splicing endonuclease.

Although *T. acidophilum* has been investigated from various viewpoints as described above, there is little knowledge about tRNA modifications, with the exception of some early studies [24,25,26,27] and our more recent work [28]. In 1981 and 1982, the sequences of the initiator ([25] and Figure 1A) and elongator tRNA^Met^ ([24] and Figure 1B) were determined. A novel modification at position 15 (N in Figure 1B), which was named later as archaeosine at position 15 (G^+^15) [29], and the typical archaeal tRNA modification of 2'-*O*-methylcytidine at position 56 (Cm56) [30] were reported. In 1991, Edmonds *et al*. [27] reported that a mixture of tRNAs from *T. acidophilum* contains *N*^7^-methylguanine (m^7^G). In general, the m^7^G modification is found at position 46 in class I tRNAs from eubacteria and eukaryotes [31,32,33,34,35]; class I tRNAs are defined as tRNAs with a variable region of regular size. To verify the location of the m^7^G modification in the tRNA, we analyzed tRNA modifications in *T. acidophilum* [28]. Unexpectedly, we found that the m^7^G modification was present at a novel position, nucleotide 49 in class II tRNA^Leu^ (Figure 1C); class II tRNAs have a long variable region. Furthermore, we found several distinct modifications in this tRNA^Leu^ (Figure 1C): 4-thiouridine at position 9 (s^4^U9) [36,37,38,39], G^+^13 [29,40], and 5-carbamoylmethyluridine at position 34 (ncm^5^U34) [41,42]. The modifications s^4^U9 and ncm^5^U34 have been not found in other archaeal tRNAs and G^+^13 has not been reported in any other tRNA [31,32]. In the current study, we tested the tRNA methyltransferase activities in crude cell extract from *T. acidophilum*, analyzed the methylated nucleosides in purified tRNAs and characterized the tRNA methyltransferases by expressing recombinant proteins in *Escherichia coli*.

## 2. Results and Discussion

### 2.1. Methylated Nucleosides in T. acidophilum tRNAs

Figure 1A–C show the cloverleaf structures of initiator tRNA^Met^, elongator tRNA^Met^ and tRNA^Leu^_UAG_, respectively. In the current study, we utilized these tRNA sequences as a basis to characterize tRNA methyltransferases in *T. acidophilum*. As shown in Table 1, various methylated nucleosides are present in these tRNAs. We predicted the candidate genes for the enzymes responsible for the modifications by BLAST searches [28] and analyzed the corresponding recombinant proteins. For example, given that the G26 in elongator tRNA^Met^ was reported to be a modified G (G*) ([24] and Figure 1B) we investigated whether recombinant Trm1 methylated G26 in the elongator tRNA^Met^ transcript. The results of the current study are summarized in Table 1. Furthermore, it should be mentioned that the sequence of the initiator tRNA^Met^ that is encoded in the genome of *T. acidophilum* strain HO-62 differs from that reported in the earlier study ([25] and Figure 1A): the nucleotide at position 57 is A instead of G in strain HO-62. In archaeal tRNAs, A57 is often modified to 1-methylinosine (m^1^I57) via 1-methyladenosine (m^1^A57) by TrmI and deamination [43,44,45]. Some possible explanations for this discrepancy are elaborated in the Discussion section. Moreover, in the case of the precursor of elongator tRNA^Met^, a standard intron is inserted at the canonical position between nucleotides 38 and 39 (Figure 1D). Therefore, it is possible that the presence of the intron might affect the methylations by tRNA methyltransferases.

**Table 1:** Methylated nucleosides in *T. acidophilum* tRNAs.

Modification and Position	Candidate Enzyme and Gene		Results from This Study
Intiator tRNA^Met^			
m^2^G26	Trm1	*Ta0997*	m^2^_2_G26 and m^2^G26 formed by Trm1.
Cm32	TrmJ?	*Ta1010m?*	^1^ We did not analyze this gene product.
Cm56	Trm56	*Ta0931*	Cm56 formed by Trm56.
m^2^G57	?		^2^ This position in the tRNA gene is A57.
m^1^A58	TrmI	*Ta0852?*	^3^ We could not detect m^1^A58 formation activity in the recombinant protein.
Elongator tRNA^Met^			
G*26	Trm1	*Ta0997*	m^2^_2_G26 and m^2^G26 formed by Trm1.
Cm32	TrmJ?	*Ta1010m?*	^1^ We did not analyze this gene product.
Cm56	Trm56	*Ta0931*	Cm56 formed by Trm56.
tRNA^Leu^_UAG_			
m^2^_2_G26	Trm1	*Ta0997*	m^2^_2_G26 formed by Trm1.
m^1^G37	Trm5	*Ta0836*	m^1^G37 formed by Trm5.
m^7^G49	?	*Ta0679? +α?*	We could not obtain soluble recombinant protein.
Cm56	Trm56	*Ta0931*	Cm56 formed by Trm56.
m^1^A58	TrmI	*Ta0852?*	^3^ We could not detect m^1^A58 formation activity in the recombinant protein.

^1^ During the course of the current study, it has been reported that *Sulfolobus acidocaldarius* TrmJ generates the Cm32 modification in tRNA. The candidate gene in *T. acidophilum* was predicted by a BLAST search; ^2^ The sequence of the initiator tRNA^Met^ that is encoded in the genome of the *T. acidophilum* strain HO-62 differs from that reported in the earlier study [25] (see Figure 1A and Figure 6A): the nucleotide at position 57 is A instead of G in strain HO-62. Furthermore, the tRNA gene in the genome of strain HO-62 contains additional nucleotides, A20b and C22; ^3^ The *Ta0852* gene product was expressed in *Escherichia coli* as a soluble protein. However, we could not detect any ability to form m^1^A58; ?, the corresponding enzyme is unknown.

### 2.2. Transfer RNA Methyltransferase Activities in the Crude Cell Extract

**Figure 2:**
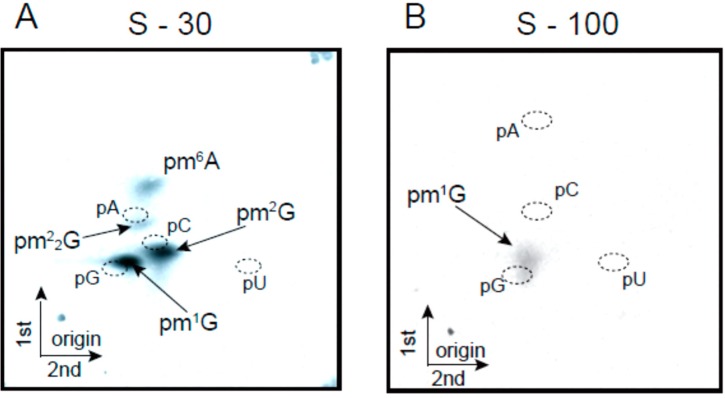
Activities of tRNA methyltransferases in the crude cell extracts. The activities of tRNA methyltransferases in the S-30 (**A**) and S-100 (**B**) fractions were analyzed by 2D-TLC. ^14^C-methylated nucleotides were monitored by autoradiography. The solvent systems were as follows: first dimension, isobutyric acid, ammonia, water, 66/1/33 *v*/*v*/*v*; second dimension, isopropyl alcohol, HCl, water, 70/15/15 *v*/*v*/*v*.

Next, we tested tRNA methyltransferase activities in crude extract from *T. acidophilum* cells. The supernatant fraction from centrifugation at 30,000× *g* (S-30) was prepared and then the tRNA^Leu^_UAG_ transcript was subjected to methylation by the S-30 extract with ^14^C-*S*-adenosyl-l-methionine (AdoMet) as the methyl group donor. The methylated tRNA was digested completely with nuclease P1 and then the resultant ^14^C-methylated nucleotides were analyzed by two-dimensional thin-layer chromatography (2D-TLC). As shown in Figure 2A, four ^14^C-methylated nucleotides (pm^1^G, pm^2^G, pm^2^_2_G and pm^6^A) could be detected. On the basis of the sequence of tRNA^Leu^_UAG_ (Figure 1C) and the candidate enzymes (Table 1), pm^1^G, pm^2^G and pm^2^_2_G, and pm^6^A were expected to be derived from the activities of Trm5, Trm1, and TrmI, respectively: pm^6^A could be converted from pm^1^A non-enzymatically [46]. However, unexpectedly, pCm and pm^7^G were not detected. In general, the formation of pCm by Trm56 is one of the most common tRNA methyltransferase activities found in crude extract from archaeal cells. For example, it was reported that Trm56 activity in relation to the formation of pCm56 is clearly detected in cell extract from *Pyrococcus furiosus* [47]. Analysis of the *T. acidophilum* proteome revealed that various proteins form several large (more than 300 kDa) protein complexes and that some protein complexes might interact with the membrane [48]. Consequently, Trm56 and an unknown tRNA (m^7^G49) methyltransferase might be included in protein complexes and precipitated by centrifugation at 30,000× *g*. We tested several buffer conditions such as variations in pH, components, detergents, and salt concentrations (data not shown). However, to date, we have not detected enzyme activities responsible for the formation of pCm and pm^7^G in crude extract from *T. acidophilum* cells, though there are other possibilities; while we used aluminum oxide to prepare the extract in this experiment, other methods for preparation of cell extract should be tested. In addition, tRNA methyltransferases in the crude extract have different affinities for ^14^C-AdoMet. The concentration of ^14^C-AdoMet in the experiment was 19.5 µM. In this case, tRNA methyltransferases, which have relatively high affinity for AdoMet, might preferentially consume the ^14^C-AdoMet. When the supernatant fraction from centrifugation at 100,000× *g* (S-100) was used as the cell extract instead of the S-30 fraction, the findings were even more marked: only the formation of pm^1^G was detectable (Figure 2B). Given that tRNA methyltransferases have a general affinity for RNA, the enzymes often bind to ribosomes and are precipitated by centrifugation at 100,000× *g*. In fact, the majority of TrmI from *Thermus thermophilus* [49] is precipitated by centrifugation at 100,000× *g* [50]. However, our findings with the extract from *T. acidophilum* are unprecedented. In the current study, we characterized tRNA methyltransferases by analyzing purified recombinant proteins. However, these enzymes might interact with other proteins and form large protein complexes in living cells.

### 2.3. Modified Nucleosides in Purified tRNAs

**Figure 3:**
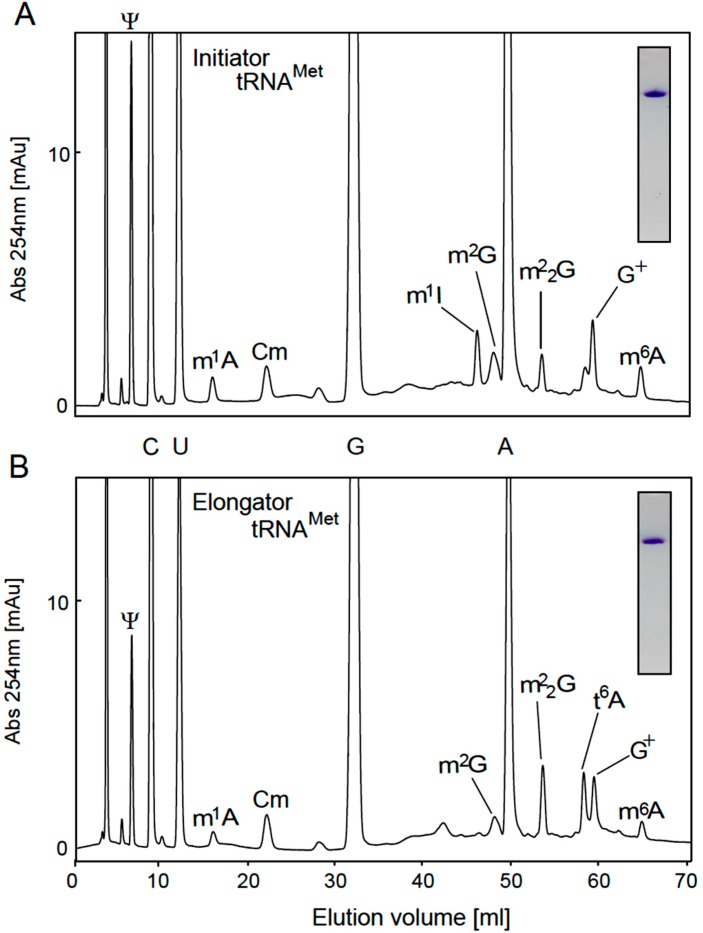
Modified nucleosides in native initiator and elongator tRNA^Met^. Native initiator and elongator tRNA^Met^ were purified by the solid-phase DNA probe method (insets). The hybridization regions of the probes are illustrated in Figure 1A,B. The modified nucleosides in the initiator (**A**) and elongator (**B**) tRNA^Met^ were analyzed by reverse phase column chromatography.

To analyze the modified nucleosides in tRNAs, we purified initiator and elongator tRNA^Met^ by the solid-phase DNA probe method [51], in which tetraalkylammonium salts were used in the hybridization buffer. Tetraalkylammonium salts destabilize the tRNA structure and enhance the formation of DNA-RNA hybrids [51], and thus we were able recently to purify single tRNA species from thermophiles such as *Aquifex aeolicus* [52], *T. thermophilus* [50,53], *Aerophyrum pernix* [54], and *T. acidophilum* [28] using this approach. In the case of initiator tRNA^Met^, because the sequence from G15 to U36 was distinct, we designed the DNA probe to this region. In the case of elongator tRNA^Met^, *T. acidophilum* two species: the sequence of one was previously determined as shown in Figure 1B [24]. These two tRNA^Met^ species differ in three nucleotides in the D-loop and anticodon arm. Therefore, we designed the DNA probe as shown in Figure 1B. As shown in the insets in Figure 3A,B, initiator and elongator tRNA^Met^ were purified successfully: the ^14^C-Met-charging activities were checked by the S-100 fraction (data not shown). The purified tRNAs were digested with snake venom phosphodiesterase, RNase A and bacterial alkaline phosphatase, and then the resultant nucleosides were analyzed by HPLC using a C18 column (Figure 3A,B). Snake venom phosphodiesterase can cleave the phosphodiester bond adjacent to 2'-*O*-methylated nucleotide. As shown in Figure 3A, m^1^A, Cm, m^1^I, m^2^G, m^2^_2_G and m^6^A were detected as methylated nucleosides in the initiator tRNA^Met^ sample. The presence of m^1^A, Cm, m^2^G and m^6^A is consistent with the published RNA sequence (Figure 1A). However, the presence of m^1^I suggests that A57 in this tRNA is modified to m^1^A57 by TrmI [44,45] and that deamination then generates m^1^I57 as in the case of *Haloferax volcanii* [43]. Furthermore, a peak for m^2^_2_G was detected, which suggests that some proportion of the initiator tRNA^Met^ contains m^2^_2_G26. Moreover, G^+^ was clearly detected, which suggests that G15 is modified to G^+^15 in initiator tRNA^Met^. In the elongator tRNA^Met^ sample, m^1^A, Cm, m^2^G, m^2^_2_G and m^6^A were detected. The modifications m^2^G and m^2^_2_G have not been reported at any position in elongator tRNA^Met^, although the uncharacterized G26 modification represents a possible location (G*26 in Figure 1B and Table 1). Consequently, the modified G26 was expected to be a mixture of m^2^G26 and m^2^_2_G26. Furthermore, *N*^6^-threonylcarbamoyladenosine (t^6^A) was also detected, which is consistent with the RNA sequence (Figure 1B). After these pilot experiments, we established expression systems in *E. coli* for the candidate genes shown in Table 1. As mentioned in Table 1, we could not obtain soluble protein from the *Ta0679* gene. Furthermore, the *Ta0852* gene product did not show TrmI activity. Consequently, these gene products were not analyzed further in the current study.

### 2.4. Formation of m^1^G37 in tRNA^Leu^_UAG_ Transcript by Ta0836 Gene Product

**Figure 4:**
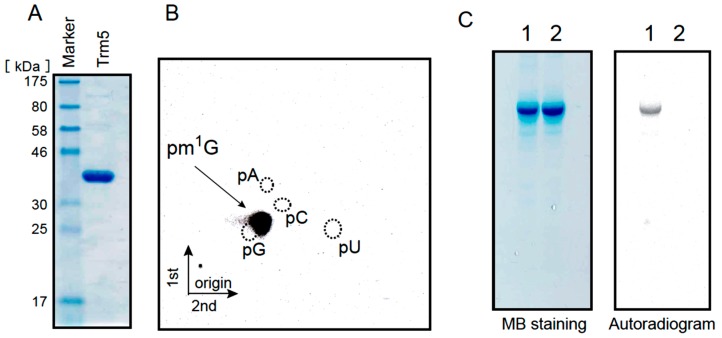
The Ta0836 gene product is *T. acidophilum* Trm5. (**A**) 10 µg of the purified *Ta0836* gene product was analyzed by 15% SDS-PAGE. The gel was stained with Coomassie Brilliant Blue; (**B**) The tRNA^Leu^_UAG_ transcript was methylated by the *Ta0836* gene product and then the generated methylated nucleotide was analyzed by 2D-TLC; (**C**) The methyl group acceptance activities of the wild-type tRNA^Leu^_UAG_ transcript (lane 1) and the mutant tRNA^Leu^_UAG_ transcript (lane 2), in which G37 was replaced by A, were investigated. The transcripts were individually incubated with the *Ta0836* gene product and ^14^C-AdoMet, and then separated by 10% PAGE (7 M urea). The gel was stained with methylene blue (**left** panel) and the autoradiogram of the same gel was taken (**right** panel).

The *Ta0836* gene product was expressed in *E. coli* and purified as shown in Figure 4A. The expected amino acid sequence of the *Ta0836* gene product shares a high degree of homology (82%) with that of the identified archaeal Trm5 (Mj0883 of *Methanocaldococcus jannaschii*) [55,56]. When the purified protein was incubated with the tRNA^Leu^_UAG_ transcript and ^14^C-AdoMet, the ^14^C-methyl group was clearly incorporated into the transcript (see Figure 4C lane 1). Analysis of the modified nucleotides by 2D-TLC revealed that the ^14^C-methylated nucleotide was pm^1^G (Figure 4B). Furthermore, when the G37 in tRNA^Leu^_UAG_ transcript was replaced by A, no methyl group incorporation was observed (Figure 4C lane 2), which indicates that the methylation site is G37. From these results, we concluded that the Ta0836 gene product is the *T. acidophilum* Trm5 protein. Given that the m^1^G modification was previously found only at position 37 in tRNA^Leu^_UAG_ in *T. acidophilum* ([28] and Figure 1C), the m^1^G modification activity in the S-100 is probably derived from Trm5.

### 2.5. Ta0997 Gene Product Is a Single Site-Specific Trm1

**Figure 5:**
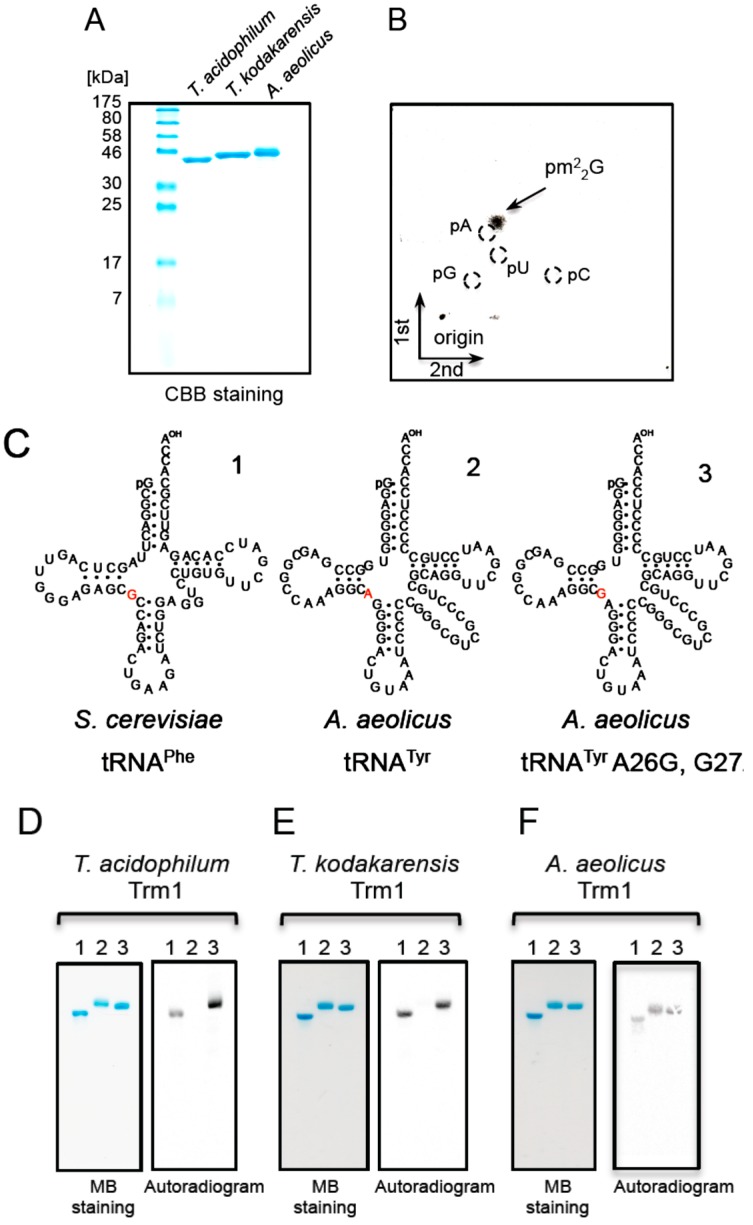
*T. acidophilum* Trm1 (the *Ta0997* gene product) is a single-site-specific enzyme. (**A**) The *T. acidophilum Ta0997* gene product (**left**), *T. kodakaraensis* Trm1 (**center**) and *A. aeolicus* Trm1 (**right**) (2 µg each) were analyzed by 15% SDS-PAGE. The gel was stained with Coomassie Brilliant Blue; (**B**) The tRNA^Leu^_UAG_ transcript was methylated by the *Ta0997* gene product and then the generated methylated nucleotide was analyzed by 2D-TLC; (**C**) The sequences of *S. cerevisiae* tRNA^Phe^, *A. aeolicus* tRNA^Tyr^ and *A. aeolicus* tRNA^Tyr^ A26G, G27A are depicted by cloverleaf structures. These tRNA transcripts were previously used to determine the site specificity of *A. aeolicus* Trm1, which is a multi-site-specific Trm1. The nucleotides at position 26 in the tRNA transcripts are colored red. The transcript numbers (1, 2, and 3) correspond to the lane numbers in panels (**D**–**F**). (**D**) The tRNA transcripts (0.1 A260 units each) were incubated with the *Ta0997* gene product (*T. acidophilum* Trm1) and ^14^C-AdoMet at 50 °C for 5 min, separated by 10% PAGE (7 M urea), and then the gel was analyzed by autoradiography. The left and right panels show the gel stained with methylene blue and its autoradiogram, respectively. *T. kodakaraensis* (**E**) and *A. aeolicus* (**F**) Trm1 proteins were analyzed by the same method.

The m^2^_2_G modification was observed only at position 26 in tRNA^Leu^_UAG_ ([28] and Figure 1C). Trm1 transfers two methyl groups to the 2-amino group in the target guanine and m^2^G is formed as an intermediate [57,58]. Consequently, the m^2^G and m^2^_2_G modifications in tRNA^Leu^_UAG_ transcript by the S-30 (Figure 2A) were expected to be derived from Trm1 activity. Trm1 enzymes can be divided into two types on the basis of their specificity for the target guanosine(s). One is a single-site-specific Trm1, which modifies only G26 and is found in eukaryotes and archaea [57,58,59,60]. The second is a multi-site-specific Trm1, which modifies both G26 and G27 and is found in the hyperthermophilic eubacterium,* A. aeolicus* [52]. In addition to the *Ta0997* gene product (Figure 5A lane 1), we prepared two types of Trm1 enzyme from *Thermococcus kodakarensis* (Figure 5A lane 2) and *A. aeolicus* (Figure 5A lane 3) as controls. The *Ta0997* gene product methylated the tRNA^Leu^_UAG_ transcript (data not shown) and ^14^C-nucleotide analysis revealed that the modified nucleotide was pm^2^_2_G (Figure 5B). These results showed that the *Ta0997* gene product is the *T. acidophilum* Trm1 protein. To distinguish the site specificity, yeast tRNA^Phe^ and *A. aeolicus* tRNA^Tyr^ transcripts were prepared (Figure 5C). These tRNA transcripts were used previously to assess the site specificity of *A. aeolicus* Trm1 [52]. Yeast tRNA^Phe^ contains the sequence G26C27, whereas *A. aeolicus* tRNA^Tyr^ contains the sequence A26G27. In addition, the *A. aeolicus* tRNA^Tyr^ A26G, G27A mutant transcript has the sequence G26A27. As shown in Figure 5D, *T. acidophilum* Trm1 methylated yeast tRNA^Phe^ and *A. aeolicus* tRNA^Tyr^ A26G, G27A transcripts, which contain G26. In contrast, *T. acidophilum* Trm1 did not methylate the wild-type tRNA^Tyr^ transcript (Figure 5D center), which contains A26. Thus, these results demonstrate that *T. acidophilum* Trm1 is a single-site-specific Trm1, which methylates only G26. Similar to *T. acidophilum* Trm1, *T. kodakarensis* Trm1 methylated only the yeast tRNA^Phe^ and *A. aeolicus* tRNA^Tyr^ A26G, G27A transcripts (Figure 5E). In contrast, *A. aeolicus* Trm1 methylated all the transcripts (Figure 5F), which indicates that *A. aeolicus* Trm1 has multi-site specificity.

### 2.6. T. acidophilum Trm1 Can Modify G26 in Initiator tRNA^Met^ Transcript to m^2^_2_G26 via m^2^G26

**Figure 6:**
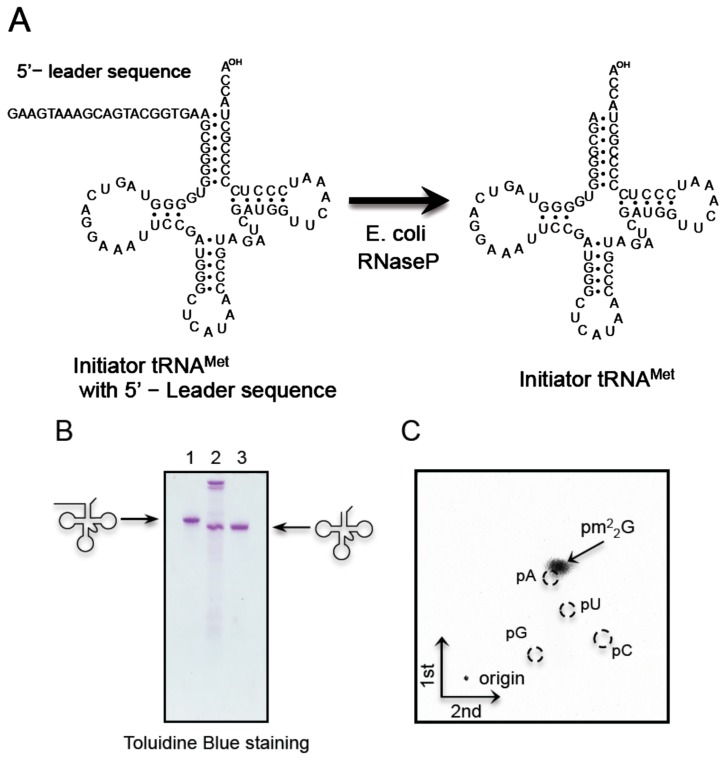
*T. acidophilum* Trm1 methylates the initiator tRNA^Met^ transcript. (**A**) Given that the 5'-end of the *T. acidophilum* initiator tRNA^Met^ is A, the transcript is not synthesized efficiently by T7 RNA polymerase. Consequently, the initiator tRNA^Met^ transcript was synthesized with a 5'-leader sequence by T7 RNA polymerase, purified by 10% PAGE (7 M urea), and then the 5'-leader sequence was removed with *E. coli* RNase P; (**B**) The cleavage of the 5'-leader sequence was monitored by 10% PAGE (7 M urea). The gel was stained with toluidine blue. The lanes are as follows: **left**, initiator tRNA^Met^ with the 5'-leader sequence; center, the reaction mixture; **right**, purified mature initiator tRNA^Met^ transcript. The M1 RNA in RNase P can be observed at the top of the gel in the center lane; (**C**) The methylated nucleotide was analyzed by 2D-TLC.

The G26 modification in initiator tRNA^Met^ was reported to be m^2^G ([25] and Table 1). Consequently, we investigated whether *T. acidophilum* Trm1 can modify G26 in the initiator tRNA^Met^ transcript to m^2^_2_G. Given that the 5'-end of initiator tRNA^Met^ is an A, T7 RNA polymerase did not synthesize the transcript efficiently. Consequently, the initiator tRNA^Met^ transcript was synthesized with a 5'-leader sequence (Figure 6A) by T7 RNA polymerase (Figure 6B, lane 1) and then the 5'-leader sequence was removed with *E. coli* RNase P ([61] and Figure 6B, lane 2). The initiator tRNA^Met^ transcript was then purified by 10% polyacrylamide gel electrophoresis in the presence of 7 M urea (PAGE (7 M urea)), (Figure 6B lane 3). Trm1 from *T. acidophilum* efficiently methylated the initiator tRNA^Met^ transcript (data not shown), and the methylated nucleotide was pm^2^_2_G (Figure 6C). Furthermore, the modified nucleoside analysis showed that native initiator tRNA^Met^ contained m^2^_2_G (Figure 3A). Taking these results together, we conclude that initiator tRNA^Met^ from *T. acidophilum* contained the m^2^_2_G26 modification in addition to m^2^G26 and that TrmI activity was responsible for these modifications.

### 2.7. T. acidophilum Trm1 Can Methylate the Precursor of Elongator tRNA^Met^ with an Intron

**Figure 7:**
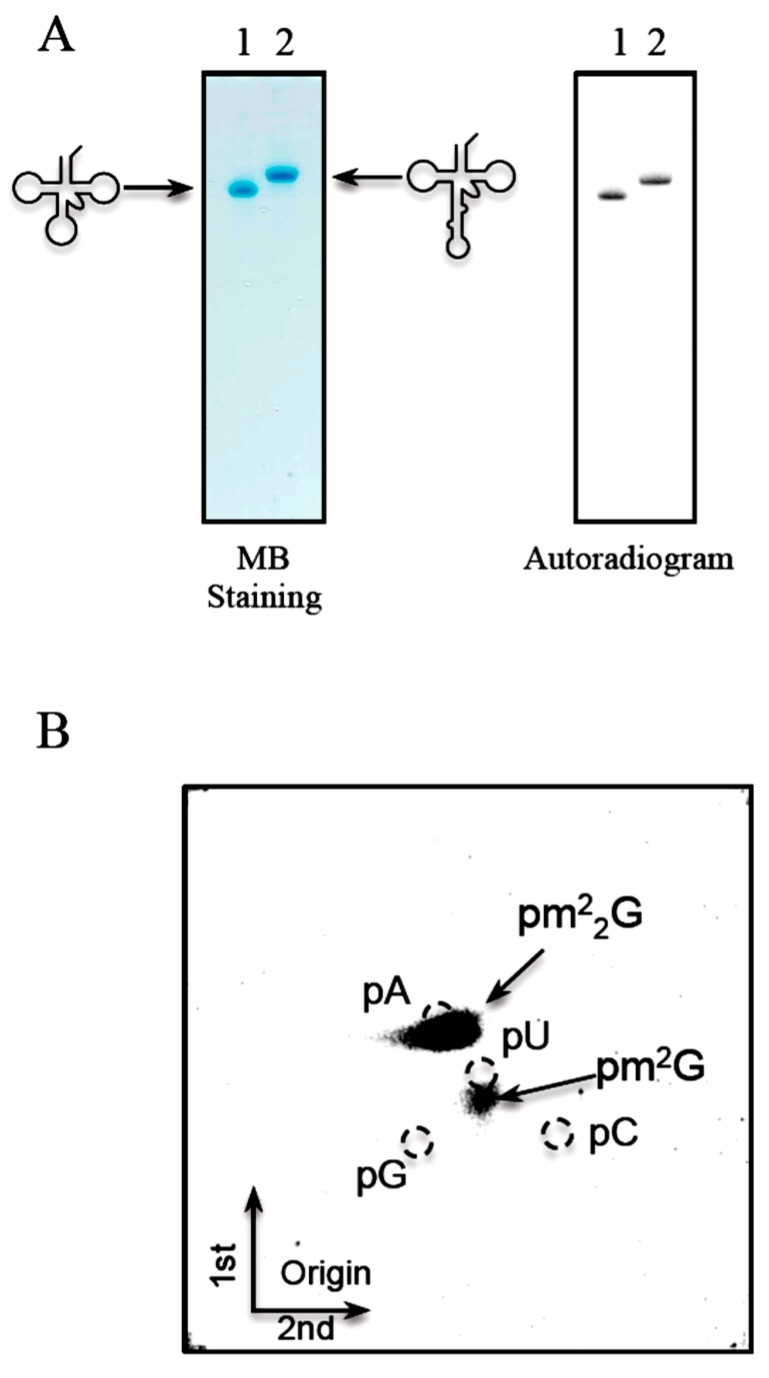
*T. acidophilum* Trm1 can methylate both the elongator tRNA^Met^ transcript and its precursor, which contains an intron. The mature transcript of elongator tRNA^Met^ and its precursor with an intron (Figure 1D) were prepared. (**A**) These transcripts were incubated with *T. acidophilum* Trm1 and ^14^C-AdoMet at 50 °C for 5 min, and then separated by 10% PAGE (7 M urea) (left panel). The gel was stained with methylene blue. The samples are as follows: lane 1, mature transcript of elongator tRNA^Met^; lane 2, precursor of elongator tRNA^Met^ with an intron. The right panel shows an autoradiogram of the same gel; (**B**) The ^14^C-methylated nucleosides in the mature transcript of elongator tRNA^Met^ were analyzed by 2D-TLC.

The G26 modification of elongator tRNA^Met^ was uncharacterized ([24], and Table 1). Furthermore, the precursor of elongator tRNA^Met^ contains an intron at the canonical position between nucleotides 38 and 39 (Figure 1D). To verify whether Trm1 could methylate the elongator tRNA^Met^ transcript and its precursor, we analyzed the methyl group acceptance activities of these RNAs (Figure 7A). Both the elongator tRNA^Met^ transcript and its precursor were methylated efficiently by *T. acidophilum* Trm1. The analysis of modified nucleotides by 2D-TLC revealed that methylated nucleotides were pm^2^G and pm^2^_2_G (Figure 7B). The analysis of modified nucleosides revealed that native elongator tRNA^Met^ contained m^2^G and m^2^_2_G (Figure 3B). Furthermore, m^2^G and m^2^_2_G modifications were not reported in the published sequence of elongator tRNA^Met^ although the modification at G26 was uncharacterized [24]. Taking these results together, we conclude that the uncharacterized G26 modification in the elongator tRNA^Met^ is a mixture of m^2^G and m^2^_2_G, which is formed by Trm1.

### 2.8. Ta0931 Gene Product Is Trm56

**Figure 8:**
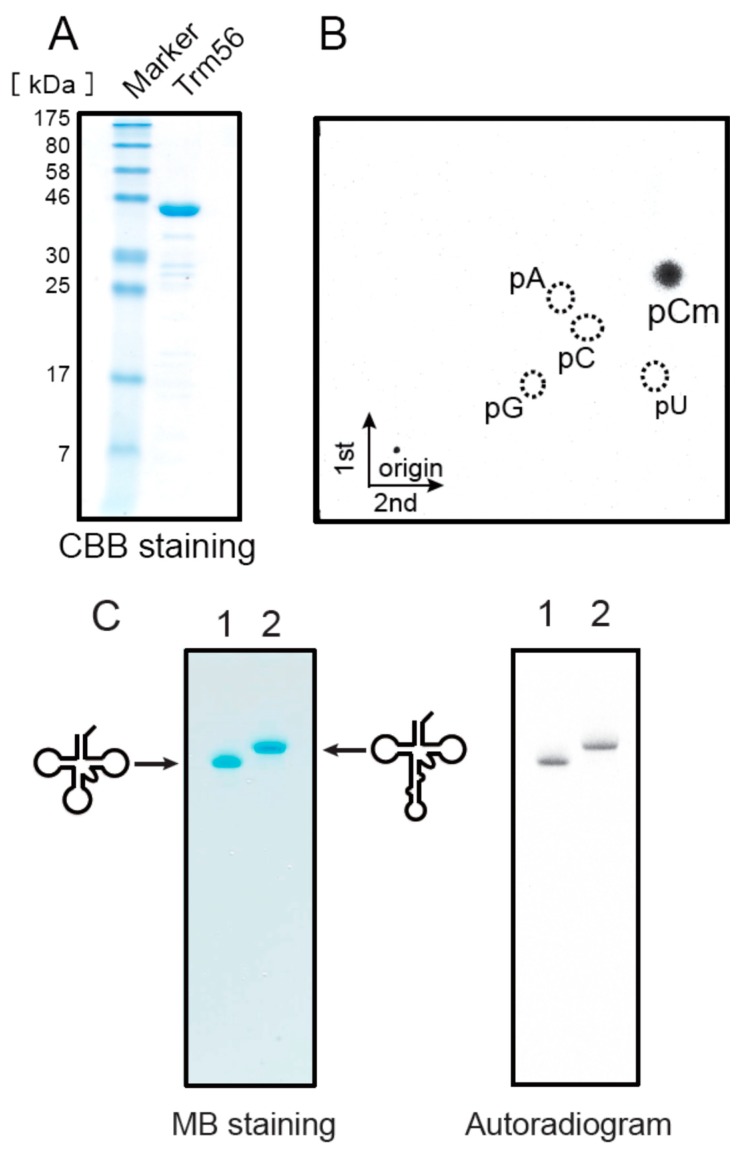

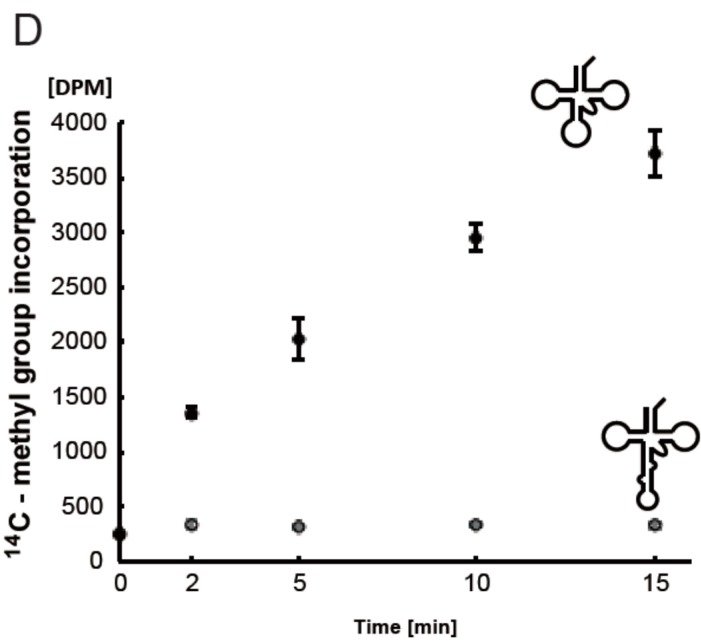
The *Ta0931* gene product (Trm56) can methylate both the elongator tRNA^Met^ transcript and its precursor with an intron. (**A**) An aliquot of 4 µg of the *Ta0931* gene product was analyzed by 15% SDS-PAGE. The gel was stained with Coomassie Brilliant Blue; (**B**) The *Ta0931* gene product and tRNA^Leu^_UAG_ transcript were incubated at 50 °C for 1 h, and then the ^14^C-methylated nucleotide was analyzed by 2D-TLC; (**C**) The incorporation of methyl groups into the mature transcript and the precursor of elongator tRNA^Met^ were investigated. The samples were as follows: lane 1, mature transcript of elongator tRNA^Met^; lane 2, precursor of elongator tRNA^Met^. The gel was stained with methylene blue (**left** panel) and the autoradiogram of the same gel was taken (**right** panel); (**D**) The incorporation of methyl groups into the mature transcript and the precursor of elongator tRNA^Met^ were measured by the filter assay. Closed and open circles show the incorporation of ^14^C-methyl groups into the mature transcript and precursor of elongator tRNA^Met^, respectively.

To analyze whether the *Ta0931* gene product was Trm56, the recombinant protein was purified as shown in Figure 8A. The purified *Ta0931* methylated the tRNA^Leu^_UAG_ transcript (data not shown) and the methylated nucleotide was identified as pCm (Figure 8B). The Cm modification is only found at position 56 in native tRNA^Leu^_UAG_ (Figure 1C). These results showed that the *Ta0931* gene product is Trm56. Neither the S-30 nor the S-100 fraction contained activity that was responsible for introducing the Cm modification into the tRNA^Leu^_UAG_ transcript (Figure 2); however, the genome does encode Trm56. This discrepancy is addressed in the Discussion section. Finally, we investigated the influence of the presence of intron on Trm56 activity. Trm56 methylated both the elongator tRNA^Met^ transcript and its precursor (Figure 8C). However, the methyl group acceptance activity of the precursor was considerably lower than that of the mature transcript (Figure 8D). It should be mentioned that the incubation in Figure 8C was performed for 12 h to show the methylation of the precursor tRNA. These results suggest that the methylation by Trm56 occurs mainly after the removal of the intron. Although the mechanism by which Trm56 recognizes tRNA has not been reported thus far, the results of the current study suggest two possibilities. The first is that the presence of the intron results in steric hindrance that prevents Trm56 binding to the substrate tRNA. The second is that Trm56 directly recognizes the anticodon loop in the tRNA. To clarify the mechanism, further study is required.

## 3. Discussion

In the current study, we investigated tRNA methyltransferase activities in crude extract from *T. acidophilum* cells, analyzed the modified nucleosides in native initiator and elongator tRNA^Met^, and characterized three tRNA methyltransferases (Trm5, Trm1 and Trm56) by purified recombinant proteins. We utilized the sequences of three tRNAs from *T. acidophilum* (initiator tRNA^Met^, elongator tRNA^Met^ and tRNA^Leu^_UAG_), which were reported previously in earlier [24,25] and our recent [28] studies, as a basis to predict the candidate genes for the enzymes responsible for the modifications. As summarized in Table 1, our experiments revealed that the genes *Ta0997*, *Ta0931*, and *Ta0836* encode Trm1, Trm56 and Trm5, respectively, from *T. acidophilum*. In archaeal tRNA modifications, there are some reports that different enzymes modify the same modification at the same position in tRNA: for example, Cm56 modification is formed by two systems, Trm56 or C/D sRNP [30]. Therefore, to understand tRNA modification systems precisely, the construction of gene disruptant mutant strains is desirable. However, there is no gene disruption method for *T. acidophilum*. Therefore, in this study, we could not utilize this approach. Consequently, the other gene product except for Trm1, Trm5 and Trm56 may bring the same modification(s) at the same position(s) in tRNA. Unexpectedly, we could not detect Trm56 activity in the crude cell extracts (S-30 and S-100 fractions). Analysis of the *T. acidophilum* proteome revealed that various proteins form several large (more than 300 kDa) protein complexes and that some of these protein complexes appear to interact with the membrane [48]. Therefore, *T. acidophilum* Trm56 might be part of a large complex with other proteins in living cells. Similar to the Trm56 activity, we could not detect tRNA (m^7^G49) methyltransferase activity in the crude cell extract. To clarify the intracellular localization of these enzymes, further study will be required. Although the recognition of tRNA by Trm56 might be affected by the presence of other proteins in the putative complex, the purified enzyme at least can act on both the mature elongator tRNA^Met^ transcript and its precursor, which contains an intron at the canonical site. The methyl group was transferred to the mature transcript much more rapidly than to the precursor tRNA with the intron. This result suggests that the methylation by Trm56 occurs mainly after the removal of the intron. The activity of Trm5 was clearly detected in the S-100 fraction and the purified recombinant Trm5 methylated the G37 nucleotide in the tRNA^Leu^_UAG_ transcript. Among the tRNA methyltransferases from *T. acidophilum*, only Trm5 seemed to act as a free enzyme, *i.e.*, was not included in a protein complex. It has been reported that Trm5 recognizes the tertiary interaction between the D- and T-arms [55,62]. Consequently, Trm5 might act mainly during and/or after the three-dimensional core of the tRNA has been reinforced structurally by the introduction of other modifications.

The sequence of the initiator tRNA^Met^ that is encoded in the genome of *T. acidophilum* strain HO-62 [3] differs from that reported in the earlier study [25]. The sequences of the two initiator tRNA^Met^ differ in the D-arm and at position 57: A20b and C22 are inserted in strain HO-62 and this strain also contains A57 instead of G57. These differences might be derived from the different origins of the strains: the strain HO-62 was isolated from Hakone, Japan [3]. There is another possibility as follows. The m^1^I 57 modification in archaeal tRNA and the initiator tRNA^Met^ gene from *T. acidophilum* were not reported in 1982. The authors used the Kuchino’s post-labelling method for tRNA sequencing [25], in which tRNA is partially cleaved by formamide and then the nucleotide at the 5'-end of each fragment is analyzed by 2D-TLC [46,63]. However, the mobility of pm^1^I on 2D-TLC closely resembles that of pm^2^G [64]. Furthermore, in general, formamide cleavage of tRNAs from thermophiles is very difficult due to their structural rigidity. Therefore, it might be difficult technically to distinguish the pm^1^I and pm^2^G on 2D-TLC. In the current study, we detected the m^1^A (m^6^A) formation into the tRNA^Leu^_UAG_ transcript by the S-30 fraction. However we could not identify the *trmI* gene, which encodes archaeal tRNA (m^1^A57/m^1^A58) methyltransferase. Consequently, we could not verify whether *T. acidophilum* TrmI can methylate the A57 in the initiator tRNA^Met^, although our analysis of modified nucleosides revealed that m^1^I is contained in the initiator tRNA^Met^. To determine the position of m^1^I modification, the RNA sequence of initiator tRNA^Met^ is required. We are now checking the plasmid vector for TrmI expression and the other gene products. From the results of the current study, we were able to add the following information with respect to initiator tRNA^Met^ from *T. acidophilum*: (1) initiator tRNA^Met^ from *T. acidophilum* strain HO-62 contains the modifications G^+^, m^1^I, and m^2^_2_G; (2) the m^2^_2_G26 modification exists in addition to m^2^G26; (3) m^2^G26 and m^2^_2_G26 are formed by Trm1.

In the current study, we have demonstrated that archaeal Trm1 can methylate the tRNA^Leu^_UAG_ transcript, which has a long variable region. As far as we know, this is the first time that archaeal Trm1 has been shown to act on class II tRNAs. In the case of class I tRNAs, archaeal Trm1 was reported to recognize the D-stem and the size of the variable region [58]. Therefore, archaeal Trm1 might be able to recognize the large variable region in the class II tRNAs. Furthermore, we showed that Trm1 efficiently methylated both the mature elongator tRNA^Met^ transcript and the precursor with an intron. These results agree well with those of a previous study [58], namely, that archaeal Trm1 does not recognize the anticodon loop. Moreover, from the results of the current study, we were able to add the following information in relation to elongator tRNA^Met^ from *T. acidophilum*: the unidentified G modification at position 26 is a mixture of m^2^G and m^2^_2_G, which is formed by Trm1.

During the course of the current study, it has been reported that *Sulfolobus acidocaldarius* TrmJ is responsible for the Cm32 modification in tRNA [65]. The Cm32 modifications in initiator and elongator tRNA^Met^ are probably formed by this new enzyme as shown in Table 1.

## 4. Experimental Section

### 4.1. Materials

[Methyl-^14^C]-AdoMet (2.14 GBq/mmol) was purchased from PerkinElmer (Tokyo, Japan). Q-Sepharose Fast Flow, HiTrap Q-Sepharose, HiTrap SP-Sepharose, HiTrap Heparin-Sepharose and HiLoad 16/600 Superdex 200 prep grade were bought from GE Healthcare Japan (Tokyo, Japan). DNA oligomers were synthesized by Invitrogen Japan (Tokyo, Japan). Other chemical reagents were of analytical grade.

### 4.2. Strain, Medium, and Culture

The culture source of *T. acidophilum* HO-62 was a gift from Prof. Akihiko Yamagishi (Tokyo University of Pharmacy and Life Sciences, Hachioji, Japan). The culture was performed at 56 °C under microaerophilic conditions as described previously [3]. The culture medium contained following components (in grams per liter): yeast extract, 1.0; Casamino acid, 1.0; (NH_4_)_2_SO_4_, 1.3; NaCl, 0.2; KH_2_PO_4_, 0.3; MgSO_4_, 0.25; CaCl_2_, 0.05; pH was adjusted to 1.8 with H_2_SO_4_. Cells in late log-phase were used for the experiments.

### 4.3. Preparation of S-30 and S-100 Fractions, and Detection of tRNA Methyltransferase Activities

Wet cells (0.3 g) were suspended in 2 mL of buffer A (50 mM Tris-HCl (pH 7.6), 5 mM MgCl_2_, 6 mM 2-mercaptoethanol, and 50 mM KCl). The cells were ground in a mortar with 0.15 g aluminum oxide and then the suspension was centrifuged at 8000× *g* for 20 min. The supernatant fraction was centrifuged further at 30,000× *g* for 2 h. The resultant supernatant fraction was used as the S-30 fraction. The S-100 fraction was the supernatant fraction by that was obtained after centrifugation at 100,000× *g* for 2 h. Transfer RNA methyltransferase activities in the S-30 and S-100 fractions were analyzed as follows: 30 µg of protein from the S-30 or S-100 fraction, 0.2 A_260_ units tRNA^Leu^_UAG_ transcript and 0.78 nmol [methyl-^14^C]-AdoMet were incubated in 40 µL of buffer A at 55 °C for 1 h. The RNA was extracted with phenol-chloroform and then recovered by ethanol precipitation. The RNA pellet was dissolved in 3 µL of 50 mM sodium acetate (pH 5.0), and digested with 2.5 units of nuclease P1 (Wako Pure Chemicals, Osaka, Japan). The sample was separated using 2D-TLC as described previously [64]. The ^14^C-methylated nucleotides were monitored with a BAS 2000 Bio-imaging Analyzer (Fuji Photo Film, Tokyo, Japan).

### 4.4. Purification of Initiator and Elongator tRNA^Met^ by the Solid-Phase DNA Probe Method

Initiator and elongator tRNA^Met^ were purified by the solid-phase DNA probe method as described in our previous reports [51,52]. The sequences of the 3'-biotinylated DNA oligomers were as follows: for initiator tRNA^Met^, 5'-ATG AGC CCA TTG GGA TTT CCT GA-biotin 3'; for elongator tRNA^Met^, 5'-ATG AGT CCG GTG CTC CTC CAG-biotin 3'. The complementary regions are illustrated in Figure 1A,B. The isolated tRNAs were further purified by 10% PAGE (7 M urea).

### 4.5. Nucleoside Analysis

Nucleoside analysis was performed as described in our previous reports [50,53]. The standard marker of G^+^ was kindly provided by Prof. Takashi Yokogawa (Gifu University, Gifu, Japan).

### 4.6. Selection of Candidate Genes

We searched for the candidate genes in the *T. acidophilum* HO-62 genome by performing a BLAST search using the amino acid sequences of *H. volcanii* Trm1 and Trm56, and *M. jannaschii* Trm5. The identification of the other candidate genes was reported in our previous paper [28].

### 4.7. Cloning of the Candidate Genes, and Expression of Gene Products

#### 4.7.1. Cloning of *Ta0836* (trm5)

The *Ta0836* gene was amplified by the polymerase chain reaction (PCR) from genomic DNA from *T. acidophilum* using the following primers: Ta Trm5F, 5'-GAG ATA TAC ATA TGC CTC CAA AGA AGT TCG TTA-3'. Ta Trm5R, 5'-CTC GAA TTC GGA TCC TTA TTA CTA TGC CTT CTT AAG GGT CAT CG-3'.

#### 4.7.2. Cloning of *Ta0997* (trm1)

The *Ta0997* gene was amplified by PCR from the genomic DNA using the following primers: Ta Trm1F, 5'-GAG ATA TAC ATA TGA TAG TGA GGG AGG GTT CAG-3'; Ta Trm1R, 5'-CTC GAA TTC GGA TCC TTA TTA TGC CGG CGA TCG TCT GTG CA-3'.

#### 4.7.3. Cloning of *Ta0931* (trm56)

The *Ta0931* gene was amplified by PCR from the genomic DNA using the following primers: Ta Trm56F, 5'-GGA GAT ATA TAC ATA TGA TAA CCG TAC TGC GGA TAA ATC AC-3'; Ta Trm56R, 5'-CTC GAA TTC GGA TCC TTA TTA GCG TAT TTC ATC GAT ATC CAT ACC-3'.

#### 4.7.4. Expression of Gene Products

The underlined regions show restriction enzyme sites (Nde I and Bam HI). The PCR products were individually inserted individually into the multiple cloning linker of expression vector pET-30a (Novagen, Cambridge, MA, USA). The gene products were expressed in the *E. coli* BL21 (DE3) Rosetta 2 strain (Novagen) in accordance with the manufacturer’s instructions.

### 4.8. Purification of Recombinant Proteins

#### 4.8.1. Purification of Trm5

Briefly, Trm5 was purified by heat treatment at 50 °C for 30 min, followed by successive rounds of column chromatography through HiTrap Q-Sepharose, HiTrap Heparin-Sepharose, and Toyopearl CM-650M (Tosoh, Tokyo, Japan). The final eluted sample was dialyzed against buffer B (50 mM Tris-HCl (pH 7.6), 50 mM KCl, 6 mM 2-mercaptoethanol and 5% glycerol) and concentrated with a Vivaspin 15R centrifugal filter device (Sartorius Japan, Tokyo, Japan). Glycerol was added to the sample to a final concentration of 50% *v*/*v* and the sample stored at −30 °C.

#### 4.8.2. Purification of Trm1

Trm1 was purified as described previously [52].

#### 4.8.3. Purification of Trm56

Briefly, Trm56 was purified by heat treatment at 50 °C for 30 min, followed by successive rounds of column chromatography through HiTrap Q-Sepharose, HiTrap Heparin-Sepharose, and HiLoad 16/600 Superdex 200 pg. The final eluted protein was dialyzed against buffer B, and concentrated with a Vivaspin 15R centrifugal filter device. Glycerol was added to the purified protein to a final concentration of 50% *v*/*v* and the samples stored at −30 °C.

### 4.9. Measurement of tRNA Methyltransferase Activities

The transcripts were prepared by using T7 RNA polymerase and purified by Q-Sepharose column chromatography and 10% PAGE (7 M urea). The standard assay for the purified enzymes was to measure the incorporation of ^14^C-methyl groups from [methyl-^14^C]-AdoMet into the appropriate tRNA transcript. For the reaction, 66 nM enzyme, 4.25 µM transcript, and 17.3 µM [methyl-^14^C]-AdoMet were incubated in 40 µL of buffer A at 50 °C for 5 min. An aliquot (35 µL) of the reaction was then used for the filter assay. To visualize the methyl-transfer reaction, we used 10% PAGE (7 M urea) and autoradiography. Briefly, tRNA (0.1 A_260_ units) was incubated with 66 nM enzyme and 17.3 µM [methyl-^14^C]-AdoMet at 50 °C for 5 min in 40 µL of buffer A, and then loaded onto a 10% polyacrylamide gel that contained 7 M urea. The gel was stained with methylene blue or toluidine blue, and then dried. The incorporation of ^14^C-methyl groups into the tRNA was monitored with a Typhoon FLA 7000 laser scanner (GE Healthcare). The 2D-TLC was performed as follows. An aliquot of 1 µg of purified protein, 0.2 A260 units of tRNA transcript and 0.69 nmol of [methyl-^14^C]-AdoMet were incubated in 40 µL of buffer A at 55°C for 1 h. The RNA was extracted with phenol-chloroform and then recovered by ethanol precipitation. The RNA pellet was digested with 1.5 units of nuclease P1. The sample was separated using 2D-TLC as described previously [64]. The ^14^C-methylated nucleotides were monitored with a Typhoon FLA 7000 laser scanner (GE Healthcare).

### 4.10. Preparation of E. coli RNase P and Removal of 5'-Leader Sequence

The plasmid vectors for the C5 protein and M1 RNA of *E. coli* RNase P were a gift from Prof. Takashi Yokogawa (Gifu University). The C5 protein was purified as described in the reference [61]. The M1 RNA was synthesized with T7 RNA polymerase. Active RNase P was generated by mixing C5 protein and M1 RNA in accordance with the method described in the reference [61]. The 5'-leader sequence of *E. coli* tRNA^Met^f was used. The 5'-leader sequence was cleaved by RNase P in accordance with the reference [61].
